# Efficient isolation of specific genomic regions retaining molecular interactions by the iChIP system using recombinant exogenous DNA-binding proteins

**DOI:** 10.1186/s12867-014-0026-0

**Published:** 2014-11-27

**Authors:** Toshitsugu Fujita, Hodaka Fujii

**Affiliations:** Chromatin Biochemistry Research Group, Combined Program on Microbiology and Immunology, Research Institute for Microbial Diseases, Osaka University, Suita, Osaka Japan

**Keywords:** iChIP, Locus-specific ChIP, r3xFNLDD-D, ChIP, Chromatin immunoprecipitation

## Abstract

**Background:**

Comprehensive understanding of mechanisms of genome functions requires identification of molecules interacting with genomic regions of interest *in vivo*. We previously developed the insertional chromatin immunoprecipitation (iChIP) technology to isolate specific genomic regions retaining molecular interactions and identify their associated molecules. iChIP consists of locus-tagging and affinity purification. The recognition sequences of an exogenous DNA-binding protein such as LexA are inserted into a genomic region of interest in the cell to be analyzed. The exogenous DNA-binding protein fused with a tag(s) is expressed in the cell and the target genomic region is purified with antibody against the tag(s). In this study, we developed the iChIP system using recombinant DNA-binding proteins to make iChIP more straightforward than the conventional iChIP system using expression of the exogenous DNA-binding proteins in the cells to be analyzed.

**Results:**

In this system, recombinant 3xFNLDD-D (r3xFNLDD-D) consisting of the 3xFLAG-tag, a nuclear localization signal (NLS), the DNA-binding domain plus the dimerization domain of the LexA protein, and the Dock-tag is used for isolation of specific genomic regions. r3xFNLDD-D was expressed using a silkworm-baculovirus expression system and purified by affinity purification. iChIP using r3xFNLDD-D could efficiently isolate the single-copy chicken *Pax5* (c*Pax5*) locus, in which LexA binding elements were inserted, with negligible contamination of other genomic regions. In addition, we could detect RNA associated with the c*Pax5* locus using this form of the iChIP system combined with RT-PCR.

**Conclusions:**

The iChIP system using r3xFNLDD-D can isolate specific genomic regions retaining molecular interactions without expression of the exogenous DNA-binding protein in the cell to be analyzed. iChIP using r3xFNLDD-D would be more straightforward and useful for analysis of specific genomic regions to elucidate their functions as compared to the previously published iChIP protocol.

**Electronic supplementary material:**

The online version of this article (doi:10.1186/s12867-014-0026-0) contains supplementary material, which is available to authorized users.

## Background

Genome functions are mediated by various molecular complexes in the context of chromatin [1]. Comprehensive understanding of mechanisms of genome functions requires identification of molecules interacting with genomic regions of interest *in vivo*. To this end, we recently developed the locus-specific chromatin immunoprecipitation (ChIP) technologies consisting of insertional ChIP (iChIP) [2–5] and engineered DNA-binding molecule-mediated ChIP (enChIP) [6–8] to isolate genomic regions of interest retaining molecular interactions. The functions of the genomic regions can be comprehensively understood by analysis of DNA, RNA, proteins, or other molecules interacting with the genomic regions.

In principle, iChIP is based on locus-tagging by inserting recognition sequences of an exogenous DNA-binding protein to isolate specific genomic regions using the exogenous DNA-binding molecule. In contrast, enChIP is based on recognition of endogenous DNA sequences by engineered DNA-binding molecules such as transactivator-like (TAL) proteins and the clustered regularly interspaced short palindromic repeats (CRISPR) system. The scheme of iChIP is as follows: (i) The recognition sequences of an exogenous DNA-binding protein such as a bacterial protein, LexA, are inserted into the genomic region of interest in the cell to be analyzed. (ii) The DNA-binding domain (DB) of the exogenous DNA-binding protein is fused with a tag(s) and a nuclear localization signal(s) (NLS(s)) and expressed in the cell to be analyzed. (iii) The resultant cell is stimulated and crosslinked with formaldehyde or other crosslinkers, if necessary. (iv) The cell is lysed, and the chromatin DNA is fragmented by sonication or enzymatic digestion. (v) The complexes including the exogenous DB are immunoprecipitated with antibody (Ab) against the tag(s). (vi) The isolated complexes which retain molecular interactions are reverse crosslinked, if necessary, and subsequent purification of DNA, RNA, proteins, or other molecules allows their identification and characterization. We successfully identified proteins and RNA components of an insulator, which functions as boundaries of chromatin domains [9], by using iChIP combined with mass spectrometry (iChIP-MS) or RT-PCR (iChIP-RT-PCR) [3]. iChIP has also been used for identification of proteins or DNA interacting with specific genomic regions by other researchers [10–13]. Thus, iChIP is a useful technology for elucidation of molecular mechanisms of genome functions.

**Figure 1:**
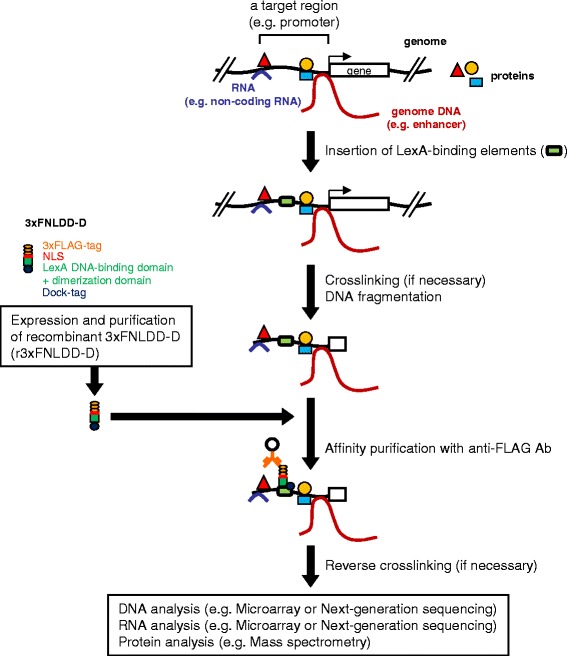
**Scheme of iChIP using r3xFNLDD-D.** 3xFNLDD-D consisting of 3xFLAG-tag, a nuclear localization signal (NLS), the DNA-binding domain (DB) plus the dimerization domain of the LexA protein, and Dock-tag, is expressed and purified. The recognition sequences of the LexA protein (LexA BE) are inserted into a genomic region of interest, usually by homologous recombination, in the cell to be analyzed. The resultant cell is stimulated and crosslinked with formaldehyde or other crosslinkers, if necessary. The cell is lysed, and the genomic DNA is fragmented. The target genomic region is affinity purified with r3xFNLDD-D conjugated with anti-FLAG antibody (Ab). After reverse crosslinking, if necessary, purification of the chromatin components (DNA, RNA, proteins, other molecules) allows their identification and characterization.

We recently developed 3xFNLDD, the second-generation tagged LexA DB consisting of 3xFLAG-tag, an NLS, and DB plus the dimerization domain of LexA, to utilize in iChIP [4]. 3xFNLDD is expressed in the cell to be analyzed for binding to the inserted LexA BE and subsequent purification of target genomic regions in the iChIP technology. If target genomic regions inserted with LexA BE can be pulled down using recombinant 3xFNLDD conjugated to Ab against the tag (Figure 1), expression of 3xFNLDD in the cell to be analyzed would not be necessary. In addition, it is not necessary to consider unexpected side effects of expression of 3xFNLDD on cell behavior, if any, making the procedure more straightforward than the conventional iChIP system using expression of the exogenous DNA-binding proteins in the cells to be analyzed.

In this study, we developed the iChIP system using the recombinant C-terminally Dock-tagged 3xFNLDD (r3xFNLDD-D). r3xFNLDD-D was expressed using a silkworm-baculovirus expression system and purified by affinity purification. iChIP using r3xFNLDD-D could effectively isolate the single-copy chicken *Pax5* (c*Pax5*) locus from a chicken B cell line, DT40. In addition, we could detect RNA associated with the c*Pax5* locus using this form of the iChIP system combined with RT-PCR. Thus, iChIP using r3xFNLDD-D would be more straightforward and useful than the conventional iChIP system using expression of the exogenous DNA-binding proteins in the cells to be analyzed to isolate specific genomic regions for their biochemical analysis.

## Results and discussion

### Expression and purification of r3xFNLDD-D

**Figure 2:**
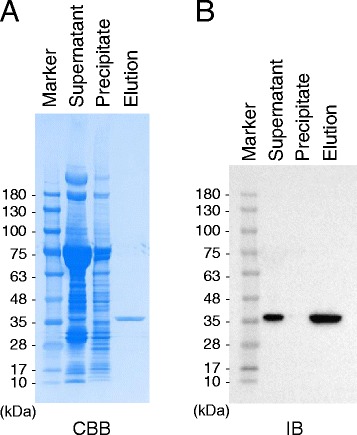
**Expression and purification of r3xFNLDD-D. (A)** Coomassie Brilliant Blue (CBB) staining of the recombinant protein. The purified proteins were subjected to SDS-PAGE and CBB staining. **(B)** Immunoblot analysis (IB) of r3xFNLDD-D. The purified proteins were subjected to SDS-PAGE and IB with anti-Dock Ab. Supernatant: the supernatant prepared from the silkworm pupal homogenates. Precipitant: the insoluble precipitate prepared from the silkworm pupal homogenates. Elution: the eluate after affinity purification with Dock Catch Resin.

For preparation of the purified r3xFNLDD-D, we utilized a silkworm-baculovirus expression system [14]. In this system, r3xFNLDD-D was expressed in a silkworm pupa by infection of baculoviruses expressing r3xFNLDD-D. The expressed protein was purified from the pupal homogenates using Dock Catch Resin, which specifically binds to Dock-tag in a calcium-dependent manner [14]. As shown in Figure 2A, SDS-PAGE followed by Coomassie Brilliant Blue (CBB) staining detected a single protein band at 35 kDa in the elution fraction. This protein was confirmed as r3xFNLDD-D by immunoblot analysis with anti-Dock Ab (Figure 2B). Thus, r3xFNLDD-D could be expressed in a silkworm pupa and purified without visible degradation.

### Efficient isolation of a target genomic region by iChIP using r3xFNLDD-D

**Figure 3:**
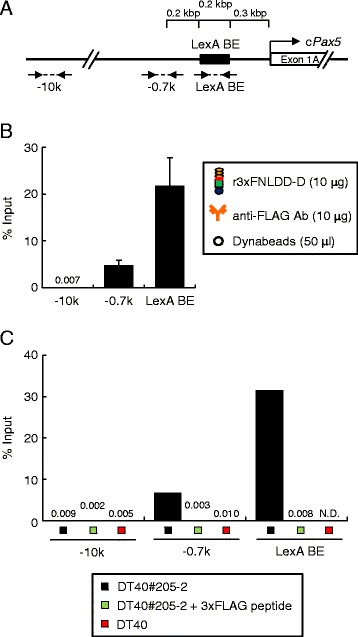
**Isolation of the c** ***Pax5*** **1A promoter region by iChIP using r3xFNLDD-D. (A)** Scheme of the LexA BE-inserted c*Pax5* 1A promoter region with primer positions. The positions of PCR primers with distances from the transcription start site (TSS) are indicated. **(B)** The results of iChIP using 10 μg of r3xFNLDD-D. % of input is shown (mean +/− SEM, n = 3). **(C)** Specific isolation of the target genomic region by iChIP using r3xFNLDD-D. N.D.: not detected.

Next, we examined whether the purified r3xFNLDD-D could be utilized for isolation of genomic regions of interest from vertebrate cells. To this end, we used the chicken DT40-derived cell line, DT40#205-2, in which 8 × repeats of LexA BE were inserted 0.3 kbp upstream of the exon 1A of the single-copy endogenous c*Pax5* gene [15] (Figure 3A). The crosslinked chromatin prepared from the cell line was subjected to iChIP using r3xFNLDD-D as shown in Figure 1. After purification of the immunoprecipitated genomic DNA, the yield of the c*Pax5* 1A promoter region was evaluated by detection of the LexA BE site (LexA BE) and the region 0.2 kbp upstream of LexA BE (i.e., 0.7 kbp upstream of the transcription start site (TSS) of c*Pax5* exon 1A) (−0.7 k) by real-time PCR (Figure 3A). As shown in Figure 3B, the yields of LexA BE and −0.7 k were more than 20% and 5% of input, respectively, when 10 μg of each r3xFNLDD-D and anti-FLAG Ab were used. In contrast, the yield of the genomic region 10 kbp upstream of the TSS of the exon 1A (−10 k) was less than 0.01%. These results suggested that r3xFNLDD-D can bind to LexA BE even in the crosslinked chromatin and iChIP using r3xFNLDD-D is able to specifically purify target genomic regions. The specific isolation of the c*Pax5* 1A promoter region was completely blocked when we inhibited binding of r3xFNLDD-D to anti-FLAG Ab with excessive amounts of 3xFLAG peptide (Figure 3C). The c*Pax5* 1A promoter region was not isolated when parental DT40 was used instead of DT40#205-2 (Figure 3C). These results clearly demonstrated that isolation of target genomic regions is mediated by binding of r3xFNLDD-D to LexA BE. The yield of the target genomic region by the modified iChIP system was comparable with that of the previously reported iChIP protocol [4].

### Optimization of iChIP using r3xFNLDD-D

**Figure 4:**
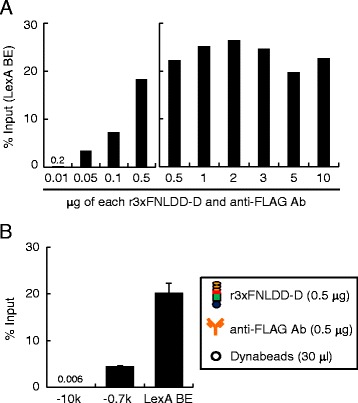
**Optimization of iChIP using r3xFNLDD-D for isolation of the c** ***Pax5*** **1A promoter region. (A)** Titration of r3xFNLDD-D and anti-FLAG Ab. **(B)** Results of iChIP using 0.5 μg of each r3xFNLDD-D and anti-FLAG Ab. % of input is shown. The error bar represents the range of duplicate experiments.

Next, we titrated amounts of r3xFNLDD-D and anti-FLAG Ab to optimize the system (Figure 4A). The yield of the LexA BE site in the c*Pax5* 1A promoter was comparable when 0.5 - 10 μg of each r3xFNLDD-D and anti-FLAG Ab was used with chromatin prepared from 1 × 10^7^ of DT40#205-2 cells. In contrast, use of 0.01 - 0.1 μg of each protein showed lower yield, suggesting that 0.5 μg of each r3xFNLDD-D and anti-FLAG Ab are sufficient for 1 × 10^7^ cells. The yield of iChIP using 0.5 μg of r3xFNLDD-D was 20% of input for LexA BE and less than 0.01% for −10 k, which is comparable with that using 10 μg of r3xFNLDD-D (Figures 3B and 4B). In this regard, we observed the yield of 15% of input for the same locus when 3xFLNDD was expressed in DT40#205-2 and the conventional iChIP protocol was used (T.F., H.F., unpublished observation). These results showed that the iChIP using r3xFNLDD-D could purify the target region with efficiency comparable to the conventional iChIP. We also examined whether it would be possible to purify the c*Pax5* 1A promoter region using r3xFNLDD-D with Dock Catch Resin, which binds to the C-terminal Dock-tag of r3xFNLDD-D. As shown in Additional file 1: Figure S1, 2% of input of the LexA BE site could be isolated with negligible contamination of −10 k, indicating that the C-terminal Dock-tag of r3xFNLDD-D can also be utilized for iChIP, although the yield was much lower than that using anti-FLAG Ab.

### Isolation of RNA associated with the c*Pax5* locus by iChIP using r3xFNLDD-D

**Figure 5:**
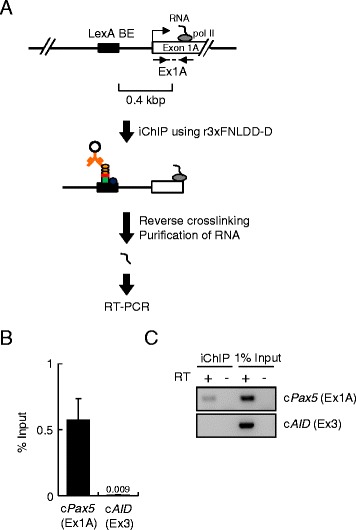
**Detection of RNA associated with the c** ***Pax5*** **locus. (A)** Scheme of iChIP. After isolation of the c*Pax5* locus by iChIP using r3xFNLDD-D, the nascent RNA transcribed on the exon 1A of the c*Pax5* gene was detected by RT-PCR. **(B)** Results of iChIP using 0.5 μg of each r3xFNLDD-D and anti-FLAG Ab. % of input is shown. The error bar represents the range of duplicate experiments. **(C)** Detection of RNA corresponding to the exon 1A of the c*Pax5* gene but not that corresponding to the exon 3 of the c*AID* gene by RT-PCR.

Next, we examined whether iChIP using r3xFNLDD-D could be utilized to isolate genomic regions of interest and identify molecules interacting with those genomic regions in cells. To this end, we attempted to isolate the c*Pax5* locus including the exon 1A region by iChIP using r3xFNLDD-D and detect the nascent RNA transcribed from the TSS of the c*Pax5* exon 1A by RT-PCR (Figure 5A). Transcription from the c*Pax5* exon 1A was not disrupted by the presence of LexA BE inserted in the 1A promoter region (Additional file 1: Figure S2). As shown in Figure 5B, iChIP using r3xFNLDD-D isolated the c*Pax5* exon 1A region but not the exon 3 region of the irrelevant c*AID* gene, which encodes an enzyme essential for B cell-specific immunoglobulin somatic hypermutation and class switch recombination [16]. After purification of the associated RNA, RT-PCR analysis detected RNA transcribed from the exon 1A of the c*Pax5* gene but not that from the c*AID* gene in the iChIP sample (Figure 5C) (the full-length images with size markers are shown in Additional file 1: Figure S3). These results suggested that iChIP using r3xFNLDD-D is able to isolate specific genomic regions retaining molecules interacting with the genomic regions.

### Feasibility of enChIP using recombinant engineered DNA-binding molecules

Lastly, we examined whether enrichment of specific genomic regions is feasible by enChIP using recombinant TAL proteins (Additional file 1: Figure S4). enChIP uses engineered DNA-binding molecules such as TAL proteins or the CRISPR system consisting of a catalytically inactive form of Cas9 (dCas9) and guide RNA (gRNA) for locus-tagging and affinity purification of the target loci [6–8]. We generated a construct expressing a fusion protein, r3xFN-5′HS5-TAL-G, consisting of 3xFLAG-tag, an NLS, a recombinant TAL protein recognizing human 5′HS5 region, which functions as an insulator to regulate transcription of the *β-globin* genes (Additional file 1: Figure S5A) [17], and the glutathione S-transferase (GST)-tag, and prepared the recombinant protein by using the silkworm-baculovirus expression system (Additional file 1: Figure S5B and C). We used the recombinant protein for enChIP analysis of the 5′HS5 locus. As shown in Additional file 1: Figure S5D, the 5′HS5 site was enriched several-fold compared to the irrelevant *interferon regulatory factor 1* (*IRF-1*) promoter region when non-crosslinked native chromatin prepared from the 293T cell line was used. These results suggest that enChIP using recombinant TAL proteins is feasible. However, we found that the r3xFN-5′HS5-TAL-G (ca. 160 kDa) showed massive degradation (Additional file 1: Figure S5B and C). In addition, we failed to detect enrichment of the target 5′HS5 site when we used crosslinked chromatin (data not shown). These results also suggest that improvement in production of recombinant TAL proteins and their access to target loci might be required for efficient isolation of target regions and identification of associated molecules.

### Applications and advantages of iChIP using r3xFNLDD-D

In this study, we applied RT-PCR to detection of RNA interacting with a genomic region of interest in cells. Next-generation sequencing or microarray analysis can be combined with iChIP using r3xFNLDD-D for non-biased identification of interacting RNA as well as DNA. Moreover, mass spectrometry can be combined for non-biased identification of interacting proteins.

Because iChIP using r3xFNLDD-D does not require expression of 3xFNLDD in cells, it is of great use in the iChIP analysis of primary cells isolated from organisms, especially higher eukaryotes such as mice. In the case of application of the standard iChIP technology to mice, it is time-consuming to establish mouse lines expressing 3xFNLDD in the cells to be analyzed as well as those possessing LexA BE in specific genomic regions. In this regard, iChIP using r3xFNLDD-D is able to skip the mating steps between mice expressing 3xFNLDD and those possessing LexA BE, substantially accelerating iChIP analysis using organisms.

Compared to enChIP or proteomics of isolated chromatin (PICh), which uses specific biotinylated nucleic acid probes such as locked nucleic acids (LNAs) that hybridize target genomic regions for their isolation [18], iChIP requires insertion of LexA BE, which takes time and effort. However, recent advancement of genome editing technologies using TALEN and CRISPR makes insertion of exogenous sequences in target loci much more easily. In addition, insertion of such exogenous sequences may abrogate function of genomic regions through changes in nucleosome positioning or other mechanisms. Therefore, it is necessary to confirm that the insertion of LexA BE does not abrogate function of genomic regions before isolating the genomic regions by iChIP. On the other hand, the locus-tagging system used in iChIP can be used for isolation of a specific target allele such as a maternal or paternal allele. Feasibility of such allele-specific analysis is one of advantages of iChIP over enChIP and PICh when allele-specific analysis is required, for example, in the analysis of genome imprinting.

## Conclusions

In this study, we established the iChIP system using r3xFNLDD-D to make the iChIP technology much more straightforward than the conventional iChIP system using expression of the exogenous DNA-binding proteins in the cells to be analyzed. Using this system, we were able to isolate target genomic regions; % of input reached more than 20% for the c*Pax5* 1A promoter region. In addition, we could detect RNA associated with the c*Pax5* locus, suggesting that iChIP using r3xFNLDD-D can isolate target genomic regions retaining molecular interactions. Thus, the modified iChIP protocol described here using r3xFNLDD-D has advantages over the previously published protocol in that it is more straightforward and useful for analysis of specific genomic regions to elucidate their functions.

## Methods

### Expression and purification of r3xFNLDD-D

Expression of 3xFNLDD-D was performed using the silkworm-baculovirus expression system (ProCube) (Sysmex Corporation, http://procube.sysmex.co.jp/eng/) as described previously [14]. Briefly, the coding sequence of 3xFNLDD [4] was inserted into the transfer vector pM31a (Sysmex Corporation) to fuse the Dock-tag at its C-terminus and co-transfected with linearized genomic DNA of ABv baculovirus (*Bombyx mori* nucleopolyhedrovirus; CPd strain, Sysmex Corporation) into the *B. mori*-derived cell line, BmN, to generate the recombinant baculovirus. The generated baculovirus was infected into a silkworm pupa to express 3xFNLDD-D. The expressed 3xFNLDD-D was purified with Dock Catch Resin (Sysmex Corporation) as described previously [14]. The immunoblot analysis was performed with anti-Dock Ab (Sysmex Corporation).

### Cell lines

The chicken B cell line DT40 was provided by the RIKEN BioResource Center through the National Bio-Resource Project of the Ministry of Education, Science, Sports and Culture of Japan. DT40 and DT40#205-2, in which LexA BE was inserted in the 1A promoter region of the c*Pax5* gene (Fujita and Fujii, manuscript submitted), were maintained in RPMI-1640 (Wako) with 4 mM glutamine, 10% (v/v) fetal bovine serum, 1% chicken serum, and 50 μM 2-mercaptoethanol at 39.5°C.

### Chromatin preparation

Cells (2 × 10^7^) were fixed with 1% formaldehyde at 37°C for 5 min. The chromatin fraction was extracted and fragmented (2 kbp-long on average) by sonication as described previously [19] except for using 800 μl of *in vitro* Modified Lysis Buffer 3 (10 mM Tris pH 8.0, 150 mM NaCl, 1 mM EDTA, 0.5 mM EGTA) and Ultrasonic disruptor UD-201 (TOMY SEIKO). After sonication, Triton X-100 was added to final concentration at 0.1%.

### iChIP using r3xFNLDD-D

The sonicated chromatin (400 μl) was pre-cleared with 0.01 - 10 μg of normal mouse IgG (Santa Cruz Biotechnology) conjugated to 30 - 50 μl of Dynabeads-Protein G (Invitrogen) and subsequently incubated with 0.01 - 10 μg of r3xFNLDD-D and anti-FLAG M2 Ab (Sigma-Aldrich) conjugated to 30 - 50 μl of Dynabeads-Protein G at 4°C for 20 h. 100 μg of 3xFLAG peptide was added to inhibit binding of r3xFNLDD-D to anti-FLAG Ab. The Dynabeads were washed four times with 1 ml of *in vitro* Wash Buffer (20 mM Tris pH 8.0, 150 mM NaCl, 2 mM EDTA, 0.1% Triton X-100) and once with 1 ml of TBS-IGEPAL-CA630 (50 mM Tris pH 7.5, 150 mM NaCl, 0.1% IGEPAL-CA630). The isolated chromatin complexes were eluted with 120 μl of Elution Buffer (500 μg/ml 3xFLAG peptide (Sigma-Aldrich), 50 mM Tris pH 7.5, 150 mM NaCl, 0.1% IGEPAL-CA630) at 37°C for 30 min. After reverse crosslinking at 65°C, DNA was purified with ChIP DNA Clean & Concentrator (Zymo Research) and used as template for real-time PCR with SYBR Select PCR system (Applied Biosystems) using the Applied Biosystems 7900HT Fast Real-Time PCR System. PCR cycles were as follows: heating at 50°C for 2 min followed by 95°C for 10 min; 40 cycles of 95°C for 15 sec and 60°C for 1 min. The primers used in this experiment are shown in Table 1.

**Table 1:** **Primers used in this study**

**Number**	**Name**	**Sequence (5′ → 3′)**	**Experiments**
26572	LexA-N2	ttctctatcgataggtacctcg	Real-time PCR in Figures 3, 4 and Additional file 1: Figure S1 (LexA BE)
27428	LexA-C-for-Pax5	cgctgcgtggtcgagcgtactg	Real-time PCR in Figures 3, 4 and Additional file 1: Figure S1 (LexA BE)
27134	cPax5-ChIP-UP(−0.2 k)-F	gggctcttatttcgtttttcttgtt	Real-time PCR in Figures 3 and 4 (−0.7 k)
27135	cPax5-ChIP-UP(−0.2 k)-R	gtgcttatttgtcagcgtggttg	Real-time PCR in Figures 3 and 4 (−0.7 k)
27013	cPax5-ChIP-UP(−10 k)-F	tccacatcgttacattgtcacttct	Real-time PCR in Figures 3, 4 and Additional file 1: Figure S1 (−10 k)
27014	cPax5-ChIP-UP(−10 k)-R	taaaagccctcagttcgatttattg	Real-time PCR in Figures 3, 4 and Additional file 1: Figure S1 (−10 k)
26552	cPax5-inExon1A-F	cctaaaacgtttagtttcagctcagt	RT-PCR in Figure 5 and Additional file 1: Figure S2 (cPax5 Ex1A)
26553	cPax5-inExon1A-R	ttcgtggctctctcaggtca	RT-PCR in Figure 5 and Additional file 1: Figure S2 (cPax5 Ex1A)
27571	cAID-Ex3-F	catgtggaggttctcttcctacg	RT-PCR in Figure 5 and Additional file 1: Figure S2 (cAID Ex3)
27572	cAID-Ex3-R	caagtttgggtaggcacgaag	RT-PCR in Figure 5 and Additional file 1: Figure S2 (cAID Ex3)
26773	18SrRNA-F2	cttagagggacaagtggcg	RT-PCR in Additional file 1: Figure S2 (18S)
26774	18SrRNA-R2	acgctgagccagtcagtgta	RT-PCR in Additional file 1: Figure S2 (18S)
27420	hHS5-TAL-Target-F	ccagtttctccagtttccctttt	Real-time PCR in Additional file 1: Figure S5 (5′HS5)
27421	hHS5-TAL-Target-R	ttttcaaaatgcaaggtgatgtc	Real-time PCR in Additional file 1: Figure S5 (5′HS5)
27310	hIRF1-prom-F	cgcctgcgttcgggagatatac	Real-time PCR in Additional file 1: Figure S5 (IRF-1)
27312	hIRF1-prom-R1 + 2	ctgtcctctcactccgccttgt	Real-time PCR in Additional file 1: Figure S5 (IRF-1)

### Isolation of interacting RNA and RT-PCR

Chromatin preparation and iChIP using r3xFNLDD-D were performed as described above except for addition of RNasin Plus RNase Inhibitor (Promega) in all buffers. After reverse crosslinking at 65°C, RNA was isolated with Isogen II (Nippon gene) combined with Direct-zol RNA Mini Prep (Zymo Research). The purified RNA was used as template for reverse transcription with ReverTra Ace qPCR RT Master Mix with gDNA Remover (Toyobo). The cDNA was used as template for PCR with AmpliTaq Gold 360 Master Mix (Applied Biosystems). PCR cycles were as follows: heating at 95°C for 10 min; 40 cycles of 95°C for 30 sec, 60°C for 30 sec, 72°C for 1 min; and the final extending 72°C for 2 min. The primers used in this experiment are shown in Table 1.

## Additional file

Additional file 1:
**Supplemental Materials.**
