# Supplementary material for: Efficient isolation of specific genomic regions retaining molecular interactions by the iChIP system using recombinant exogenous DNA-binding proteins
Source: BMC Mol Biol. 2014 Nov 27;15:26. doi: 10.1186/s12867-014-0026-0 (PMC4253623; doi:10.1186/s12867-014-0026-0)
Supplement: Additional file 1: — Supplemental Materials. [file 12867_2014_26_MOESM1_ESM.pdf]

Supplemental Materials

Efficient isolation of specific genomic regions retaining molecular interactions by the iChIP system using recombinant exogenous DNA-binding proteins

Toshitsugu Fujita and Hodaka Fujii

Inventory

Supplemental Materials and Methods

Supplemental Figure Legends

Supplemental Figures S1 - S5

Supplemental Materials and Methods

iChIP using r3xFNLDD-D with Dock Catch Resin

Cells (2×10^7) were fixed with 1% formaldehyde at 37°C for 5 min. The chromatin fraction was extracted and fragmented (2 kbp-long on average) by sonication as described previously [19] except for using 800 µl of Calcium Buffer (10 mM Tris pH 8.0, 150 mM NaCl, 2 mM $CaCl_2$) and Ultrasonic disruptor UD-201 (TOMY SEIKO). After sonication, TritonX-100 was added to final concentration at 0.1%. The sonicated chromatin (400 µl) was pre-cleared with 30 µl of mouse IgG-agarose (Sigma-Aldrich) and subsequently incubated with 30 µl of Dock Catch Resin (Sysmex Corporation) at 4°C for 20 h. Dock Catch Resin was washed four times with 1 ml of Calcium Buffer with 0.1% TritonX-100 and once with 1 ml of Calcium Buffer with 0.1% IGEPAL-CA630. The isolated chromatin complexes were eluted with 120 µl of Elution Buffer (50 mM Tris pH 7.5, 150 mM NaCl, 5 mM EGTA, 0.1% IGEPAL-CA630) at 25°C for 30 min. After reverse crosslinking at 65°C, DNA was purified with ChIP DNA Clean & Concentrator (Zymo Research) and used as template for real-time PCR with SYBR Select PCR system (Applied Biosystems) using the Applied Biosystems 7900HT Fast Real-Time PCR System.

RT-PCR

Total RNA extracted with Isogen II (Nippon gene) was used as template for reverse transcription with ReverTra Ace qPCR RT Master Mix with gDNA Remover (Toyobo). The cDNA was used as template for PCR with AmpliTaq Gold 360 Master Mix (Applied Biosystems). PCR cycles were as follows: heating at 95°C for 10 min; 15 - 35 cycles of 95°C for 30 sec, 60°C for 30 sec, 72°C for 1 min; and the final extending 72°C for 2 min. The primers used in this experiment are shown in Table 1.

Expression and purification of r3xFN-5'HS5-TAL-G

r3xFN-5'HS5-TAL-G was expressed using the silkworm-baculovirus expression system (ProCube) (Sysmex Corporation, http://procube.sysmex.co.jp/eng/) as described previously [14]. Briefly, the coding sequence of the TAL protein recognizing 5'-TGTCTATACTCACTTCCCCATTTCT-3' in the h5'HS5 region was designed (Life technologies) and fused with 3xFLAG-tag and NLS (3xFN-5'HS5-TAL) at the N-terminus. 3xFN-5'HS5-TAL was inserted into the transfer vector pM47 (Sysmex Corporation) to fuse the GST tag at its C-terminus and co-transfected with linearized genomic DNA of the ABv baculovirus (*Bombyx mori* nucleopolyhedrovirus; CPd strain, Sysmex Corporation) into the *B. mori*-derived cell line, BmN, to generate the recombinant baculovirus. The generated baculovirus was infected into a silkworm pupa to express 3xFN-5'HS5-TAL fused C-terminally with GST (3xFN-5'HS5-TAL-G). The expressed 3xFN-5'HS5-TAL-G was purified with Glutathione Sepharose 4B (GE Healthcare). Immunoblot analysis was performed with anti-FLAG M2 Ab (Sigma-Aldrich).

Cell lines

293T cell line was maintained in D-MEM (Wako) with 10% (v/v) fetal bovine serum at 37°C.

Chromatin preparation

293T cells (2×10^7) were collected and lyzed in 10 ml of Cell Lysis Buffer (10 mM Tris pH 8.0, 1 mM EDTA, 0.5% IGEPAL-CA630) at 4°C for 10 min. After centrifugation at 930 × *g* for 8 min at 4°C, the pellet was washed with 10 ml of Tris-Ca buffer (50 mM Tris pH 8.0, 1 mM

$CaCl_2$). The pellet was incubated in 1 ml of Tris-Ca buffer with 0.2% Triton X-100, 10,000 gel units of Micrococcal nuclease (New England Biolabs) and complete protease inhibitor cocktail without EDTA (Roche) at 37°C for 4 min. The reaction was stopped by adding 80 µl of 0.1 M EGTA. After centrifugation at 16,000 × *g* for 10 min at 4°C, the supernatant was collected as the chromatin fraction (average length: ca. 1 kbp).

enChIP using r3xFN-5'HS5-TAL-G

The chromatin (300 µl) was mixed with 1.1 ml of *in vitro* Modified Lysis Buffer 3 and pre-cleared with 5 µg of normal mouse IgG (Santa Cruz Biotechnology) conjugated to 30 µl of Dynabeads-Protein G (Invitrogen). The supernatant was incubated with 5 µg of 3xFN-5'HS5-TAL-G at 37°C for 20 min and mixed with Triton X-100 for 0.1% of concentration and 5 µg of anti-FLAG M2 Ab (Sigma-Aldrich) conjugated to 30 µl of Dynabeads-Protein G at 4°C for 2 h. The Dynabeads were washed four times with 1 ml of *in vitro* Wash Buffer and once with 1 ml of TBS-IGEPAL-CA630. The isolated chromatin complexes were eluted with 60 µl of Elution Buffer at 37°C for 20 min. DNA was purified with ChIP DNA Clean & Concentrator (Zymo Research) and used as template for real-time PCR with SYBR Select PCR system (Applied Biosystems) using the Applied Biosystems 7900HT Fast Real-Time PCR System. The primers used in this experiment are shown in Table 1.

Supplemental Figure Legends

Supplemental Figure S1. iChIP using r3xFNLDD-D with Dock Catch Resin.

Supplemental Figure S2. RT-PCR analysis of c*Pax5* and c*AID* mRNA. (**A** and **B**) Total RNA was extracted from DT40 and DT40#205-2 and used in RT-PCR for detection of c*Pax5* mRNA (A) and c*AID* mRNA (B). (**C**) 18S rRNA was detected as the internal control. M: Molecular size markers.

Supplemental Figure S3. The full-length images of Figure 5C including molecular size markers. c*Pax5* (Ex1A) and c*AID* (Ex3) are shown in the upper panel and lower panels, respectively. M: Molecular size markers.

Supplemental Figure S4. Scheme of enChIP using recombinant engineered DNA-binding molecules. Recombinant engineered DNA-binding molecules consisting of 3xFLAG-tag, a nuclear localization signal (NLS), the engineered DNA-binding molecule, and C-terminal tag, is expressed and purified. The r3xFN-TAL-G protein consisting of 3xFLAG-tag, NLS, a TAL protein recognizing the target sequence, and GST-tag is shown as an example of the fusion molecules. The cell to be analyzed is stimulated and crosslinked with formaldehyde or other crosslinkers, if necessary. The cell is lysed, and the genomic DNA is fragmented. The target genomic region is affinity purified with r3xFN-TAL-G conjugated to anti-FLAG antibody (Ab). After revers crosslinking, if necessary, purification of the chromatin components (DNA, RNA, proteins, other molecules) allows their identification and characterization.

Supplemental Figure S5. Isolation of the h5'HS5 region by enChIP using r3xFN-5'HS5-TAL-G. (**A**) Scheme of the 5'HS5 region with the recognition sequence of r3xFN-5'HS5-TAL-G and the position of PCR primers. (**B**) Coomassie Brilliant Blue (CBB) staining of the recombinant protein. The purified proteins were subjected to SDS-PAGE and CBB staining. Supernatant: the supernatant prepared from the silkworm pupal homogenates. Precipitant: the insoluble precipitate prepared from the silkworm pupal homogenates. Elution: the eluate after affinity purification with Glutathione Sepharose 4B. (**C**) Immunoblot analysis (IB) of r3xFN-5'HS5-TAL-G. The purified proteins were subjected to SDS-PAGE and IB with anti-FLAG Ab. (**D**) The yield of enChIP with 5 μg of r3xFN-5'HS5-TAL-G. % of input is shown. The error bar represents the range of duplicate experiments.

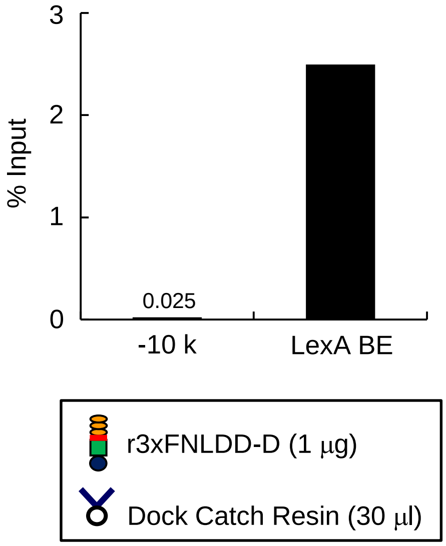

Supplemental Figure 1. Fujita and Fujii

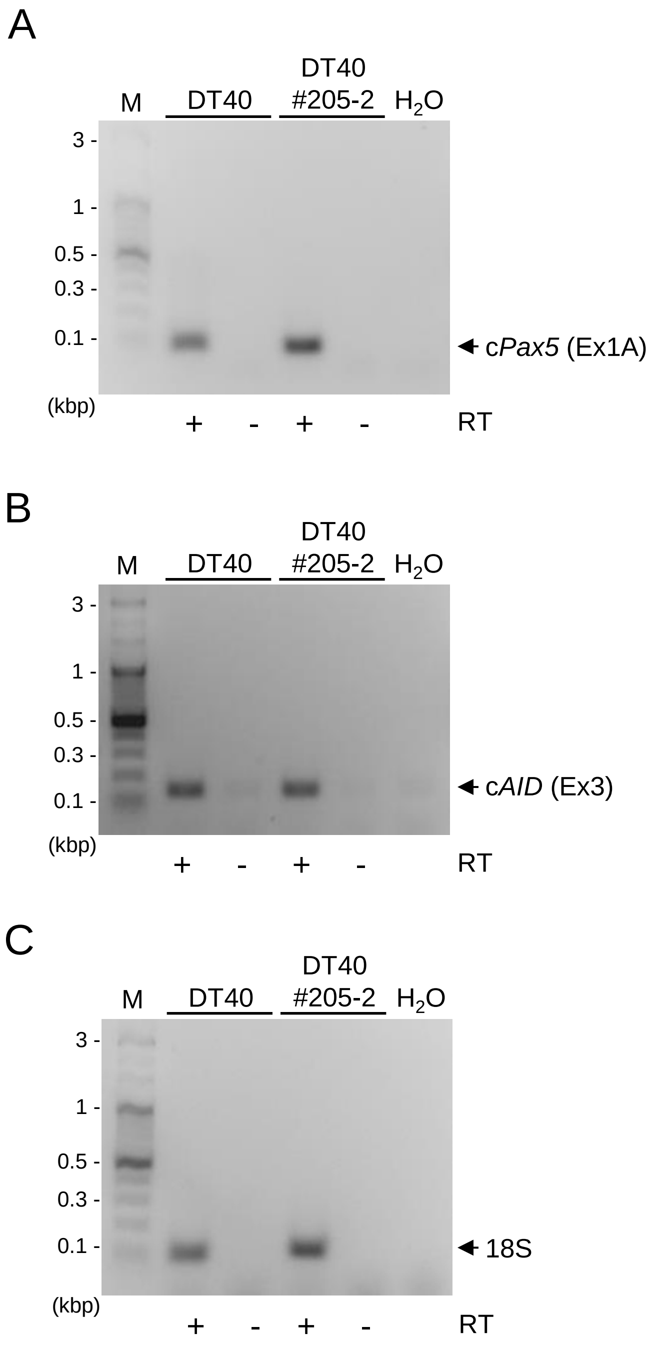

Supplemental Figure 2. Fujita and Fujii

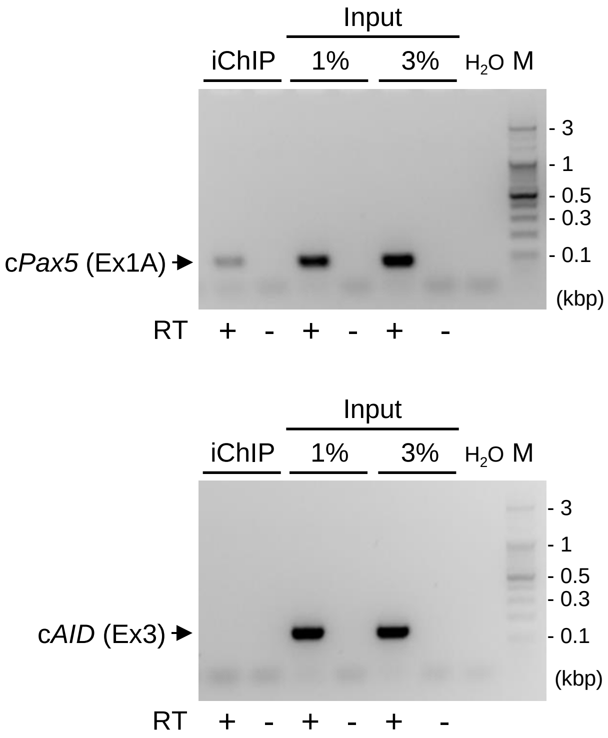

Supplemental Figure 3. Fujita and Fujii

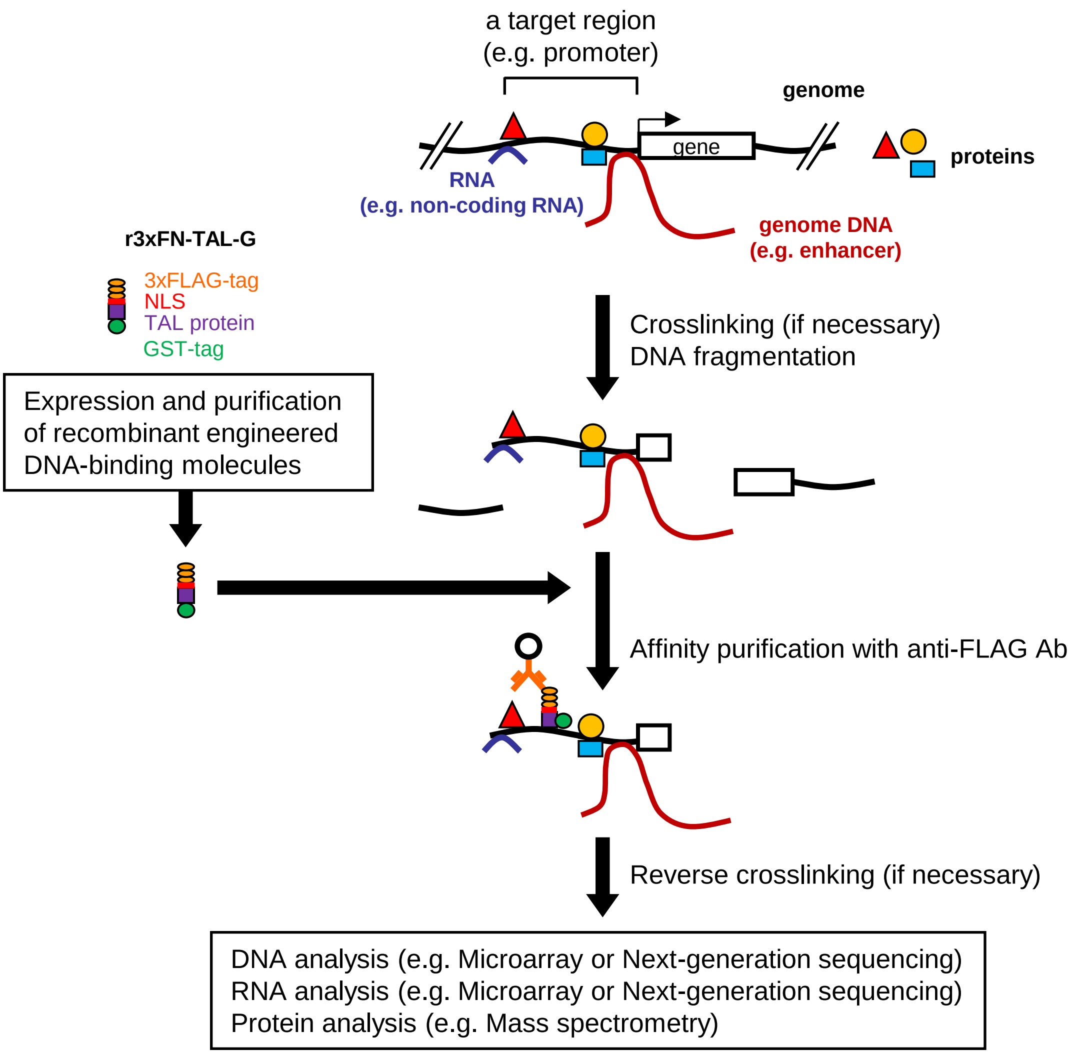

Supplemental Figure 4. Fujita and Fujii

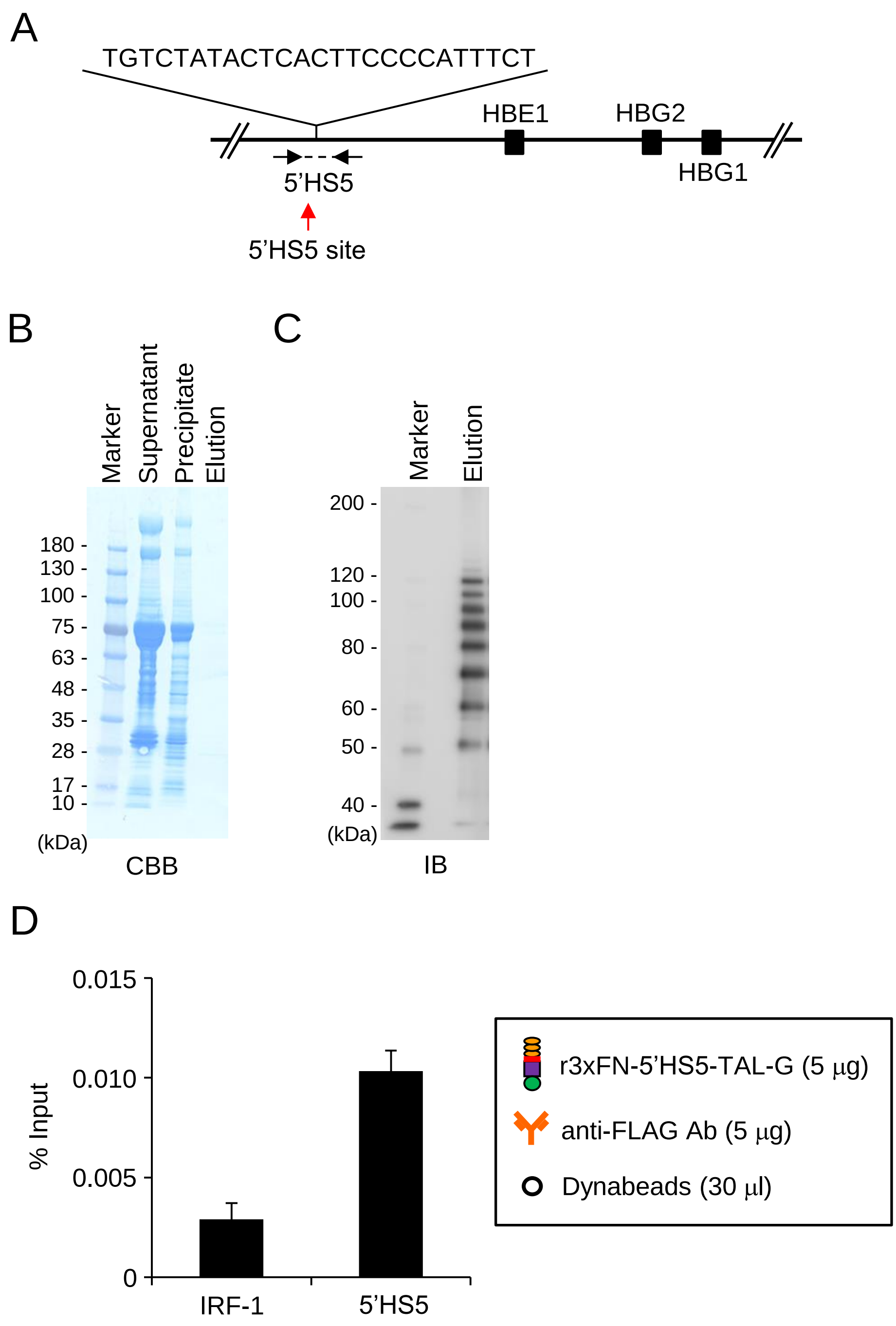

Supplemental Figure 5. Fujita and Fujii
